# Trichoepithelioma presenting as leonine facies in a young female

**DOI:** 10.1002/ski2.177

**Published:** 2022-10-19

**Authors:** Mehdi Ghahartars, Seyedeh Yasamin Parvar, Leila Samipour, Maryam Hadibarhaghtalab

**Affiliations:** ^1^ Molecular Dermatology Research Center Shiraz University of Medical Sciences Shiraz Iran; ^2^ Student Research Committee Shiraz University of Medical Sciences Shiraz Iran; ^3^ Department of Dermatology School of Medicine Kerman University of Medical Sciences Kerman Iran

## Abstract

Trichoepithelioma is a rare benign tumour of the pilosebaceous unit that originates from the hair follicles. Although it rarely results in facial disfigurement, it is thought to be the cause of leonine facies. We discuss a 27‐year‐old woman who presented with facies Leonine caused by trichoepitheliomas. The first line of treatment for these multiple symmetrical, firm, and round papules or nodules is excisional surgery.

## Graphical abstract

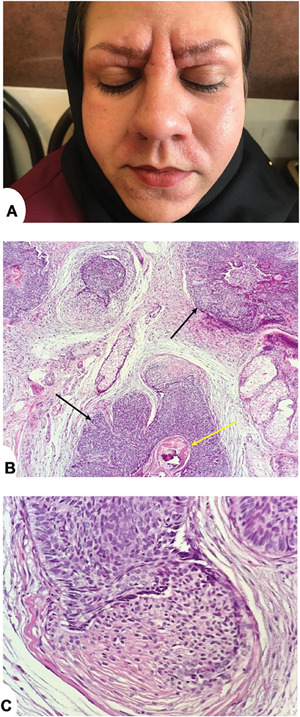

Leonine facies are rare manifestations of Trichoepithelioma where skin‐toned papules are resulting in grooves and fissures on the face. This paper describes a 27‐year‐old woman presented with a painless firm erythematous plaques. The patient presented with leonine facies and was diagnosed with Trichoepithelioma.

## 1 CASE PRESENTATION

**FIGURE 1:**
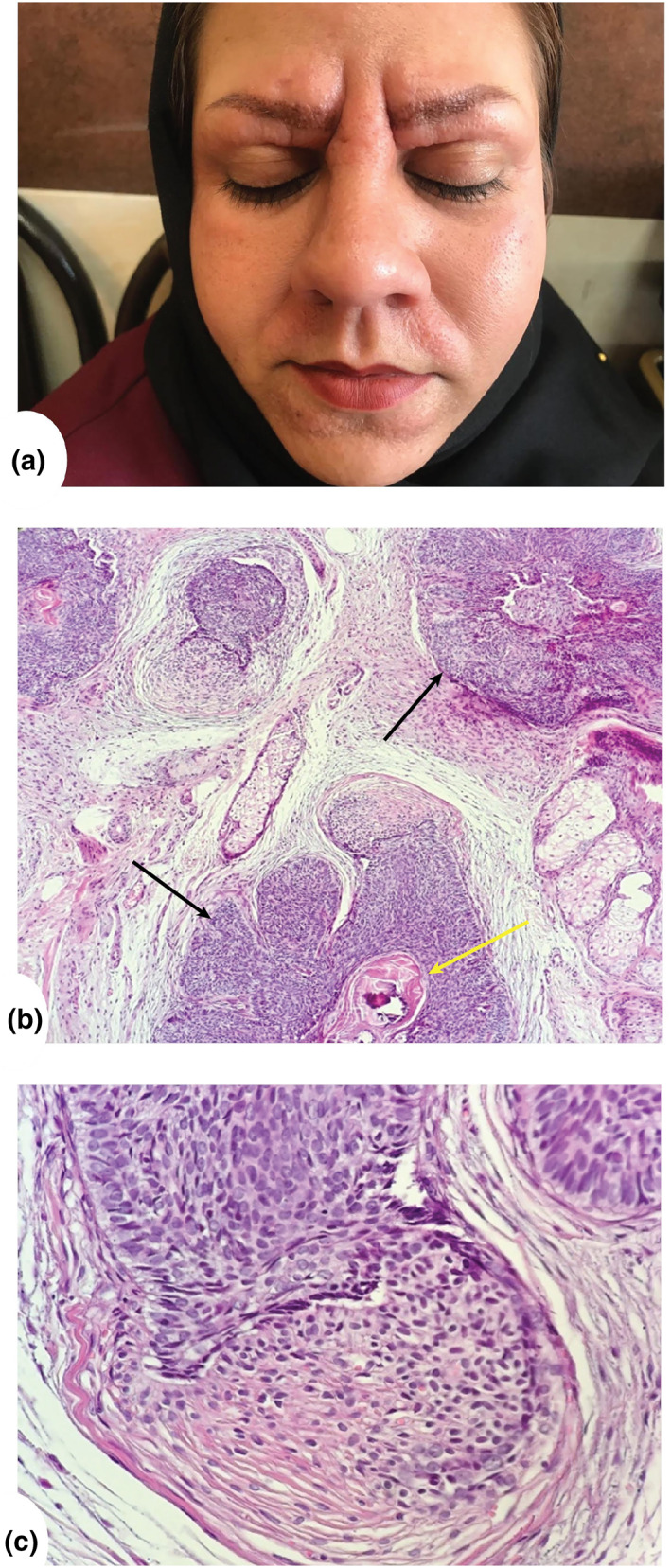
(a) Multiple aggregated skin‐coloured papules which are closely and firm. (b) Peripheral palisading in islands of basaloid cells with abortive hair papillae (black pointers) and trichilemmal keratinization (yellow pointer, ×100, H&E). (c) Abortive hair papilla (×400, H&E).

A 27‐year‐old woman, known case of hypothyroidism, with Fitzpatrick skin type III, a doubtful history of systematic lupus erythematosus, and suffering from eyebrow loss presented with a year of history of painless firm erythematous plaques to our dermatology clinic. The plaques were localized to her orbital rim, glabella, nose, nasolabial fold, and perioral area. Aggregation of the lesion across the middle of the face implies the appearance of leonine facies (Figure 1). The patient's drug history includes 200 mg hydroxychloroquine, 5 mg prednisolone, 0.1 mg levothyroxine, and a Ca‐D supplement daily for the past 2 years.

The antinuclear antibody and anti‐Ro were positive. Routine blood tests, vitamins, and other lab data were within the normal range (including hepatitis panels, anti‐La, anti‐Ribonucleoprotein, Antineutrophil Cytoplasmic Antibodies, HIV antibody, anti‐Double Strand DNA, Scleroderma‐70 antibodies, and anti‐Smith antibodies).

No similar lesions were found in the patient's first‐degree relatives. Moreover, negative history of hyper or hypopigmented and hypoaesthetic lesions was found, which practically ruled out the diagnosis of leprosy and leishmaniasis.

The patient underwent an excisional biopsy. A dermal tumour with a focal epidermal connection was discovered under the microscope. The tumour is made up of basaloid cell islands, some of which have peripheral palisading and abortive hair follicle differentiation. Evidence of trichilemmal keratinization was seen (Figure 1). According to the mentioned histopathological features, the differential diagnosis of mucinosis, mastocytosis, amyloidosis, lichen myxedematosus, cutaneous lymphoma, actinic reticulated, lipoid proteinosis, non‐langerhans cell histiocytosis, familial cylindromatosis, and Brooke‐Spiegler syndrome was excluded. The immunohistochemical profile on formalin‐fixed paraffin‐embedded eyebrow tissue revealed diffuse BCL2 positive cells, CD10 and CD34 positive cells in the stroma, and negative cytokeratin 7 marker resulting in the final diagnosis of Trichoepithelioma. Finally, the patient was followed up for more than a year, and no procedure was performed concerning her decision.

## 2 DISCUSSION

This article describes a young female diagnosed with Trichoepithelioma who presents with a leonine face. Trichoepithelioma is an uncommon benign neoplasm of the pilosebaceous unit that originates from the hair follicles. They typically manifests in females during early childhood or puberty.^1^ It was first introduced by Brooke and Fordyce in England and the United States in 1892. Its precise prevalence is not known. An American dermatopathology laboratory reported between 2.14 and 2.75 cases of trichoepitheliomas per year in 9000 cases.^2^ Despite the fact that it is an autosomal dominant disease, females are more affected, possibly due to males' smaller chromosomal expression.^3^ The disease primarily affects young people aged 10–20.^4^

Trichoepithelioma commonly presents with multiple 2–5 mm skin‐toned, firm, round, translucent, shiny, well‐defined papules and nodules. The lesions are frequently symmetrically on the face, predominantly around the nasolabial folds, forehead, nose, and eyelids. The scalp, upper trunk, and neck are less commonly affected.^2^ The lesions can fuse to form large plaques, rarely leading to grooves on the face resembling leonine facies. As far as our knowledge, there have only been three case reports of the leonine face in Trichoepithelioma. A Dongre et al. reported the first cases of leonine facies in a male patient with Trichoepithelioma in 2010.^5^ In 2016 and 2017, Bhari N and Sanjay Singh described this feature in two other men, respectively.^6^, ^7^

The lesions can be slightly depressed or umbilicated in the centre. Trichoepithelioma lesions are asymptomatic if there is no ulceration or pruritus. However, they can develop into malignant neoplasms, such as basal cell carcinoma and trichoblastic carcinoma. Physicians should scrutinize these patients more thoroughly and pay close attention to any rapid growth or ulceration.^2^

Leonine facies are a rare and morphologic manifestation of spread cutaneous infiltration of the face in which skin‐toned papules coalesce into large plaques resulting in grooves and fissures on the face.^8^ It may be progressed in size and number by age. Leonine facies can be present in a wide range of infectious and non‐infectious conditions. Granulomatous lesions like lepromatous leprosy, cutaneous sarcoidosis, leishmaniasis, chronic dermatologic disorders, and haematological malignancies, especially mycosis fungoides and leukaemia cutis, have been reported to be associated with the leonine facies.^6^, ^9^

Diagnosis is primarily grounded in clinical and histopathological features. Histologically, Trichoepithelioma represents a subset of trichoblastoma composed of smaller islands of basaloid cells and horn cysts, sometimes only one to two cells thick, with marked fibrosis. In comparison to squamous cell carcinoma, Trichoepithelioma has complete keratinization in horn pearls.^6^, ^10^

In this case, the patient refused treatment due to lack of pain and displeasure with her following the excisional biopsy. The most commonly used treatment options include excisional surgery, pharmacotherapy (such as 5% imiquimod cream), laser resurfacing, dermabrasion, electro‐surgery, and targeted therapies (such as antitumour necrosis factor‐alpha, targeting mTOR, and hypoxia signalling pathways).^2^, ^11^, ^12^, ^13^

## 3 CONCLUSION

Trichoepithelioma can manifest as leonine facies, so when confronted with this feature, trichoepithelioma should be considered among the differential diagnosis.

## CONFLICT OF INTEREST

The authors declared that they have no conflicts of interest to this work.

## AUTHOR CONTRIBUTIONS

**Mehdi Ghahartars**: Data curation (Lead); Validation (Equal). **Seyedeh Yasamin Parvar**: Investigation (Equal); Writing – review & editing (Equal). **Leila Samipour**: Investigation (Equal); Writing – original draft (Equal). **Maryam Hadibarhaghtalab**: Conceptualization (Equal); Project administration (Equal); Supervision (Equal).

## ETHICS STATEMENT

IRB have accepted the articles.

## Data Availability

The data that support the findings of this study are available on request from the corresponding author. The data are not publicly available due to privacy or ethical restrictions.
